# Pancytopenia and Hypocalcemia: An Unexpected Cause

**DOI:** 10.7759/cureus.116031

**Published:** 2026-09-10

**Authors:** Gabriela Paulo, Carolina Veiga, Marco Oliveira Dias, Ilda Coelho

**Affiliations:** 1 Internal Medicine, Unidade Local de Saude de Barcelos/Esposende, Barcelos, PRT; 2 Hematology, Unidade Local de Saúde de Braga, Braga, PRT

**Keywords:** case report, hypocalcaemia, osteoblastic lesions, pancytopenia, signet-ring cell gastric adenocarcinoma

## Abstract

Pancytopenia refers to the simultaneous reduction of all peripheral blood cell lines and is a hematologic finding often indicative of severe and potentially life-threatening disease. Although usually not associated, hypocalcemia and pancytopenia may co-occur, suggesting a common etiology.

We report a case of a caucasian woman in her late 50s with asthenia, paraesthesia and nausea. Laboratory results showed severe hypocalcemia, anemia, elevated alkaline phosphatase and lactate dehydrogenase. A computed tomography scan revealed extensive osteosclerosis, suggesting osteoblastic metastasis. Progression to pancytopenia prompted bone marrow evaluation, ultimately leading to the diagnosis of signet-ring cell gastric adenocarcinoma. Unfortunately, the patient died shortly after diagnosis. This case illustrates a rare presentation of gastric adenocarcinoma, featuring hypocalcemia due to osteoblastic metastases and pancytopenia from bone marrow infiltration - an association infrequently reported in the literature - and emphasizes that this unusual constellation of findings may be the first clue to an otherwise unsuspected solid malignancy.

## Introduction

Gastric cancer is the fifth most common malignancy and remains a major global health burden [1]. With the introduction of *Helicobacter pylori* eradication therapy, the occurrence of gastric adenocarcinoma has declined [1]. Nevertheless, there has been a rising trend in signet-ring cell carcinoma, which now represents between 8% and 30% of gastric cancer cases [1]. Common sites of distant metastasis include the peritoneum and liver [2]. In contrast, osseous and bone marrow metastases are rare, occurring in only a small percentage of cases [2,3].

We describe a case of a woman in her late 50s with hypocalcemia and anemia, which progressed to pancytopenia, leading to a bone marrow evaluation and the subsequent diagnosis of signet-ring cell gastric adenocarcinoma.

This case emphasizes the challenges in the diagnosis of signet-ring cell gastric adenocarcinoma, its uncommon manifestations and its associated poor prognosis.

## Case presentation

A woman in her late 50s presented to the Emergency Department (ED) with a three-month history of asthenia and paraesthesia around her fingertips and perioral area. She also reported nausea, but denied fever, vomiting, changes in bowel habits or overt bleeding.

She had attended the ED twice previously for similar symptoms and had recently received one unit of packed red blood cells (pRBCs) for newly diagnosed anemia (hemoglobin 7.9 g/dL). 

Past history included a total thyroidectomy eight years prior for multinodular goiter, along with a hysterectomy with bilateral salpingo-oophorectomy thirteen years ago, due to uterine leiomyoma. Her chronic medication included levothyroxine 112 mcg daily, without any dietary supplements. She had no history of alcohol or tobacco use.

On examination, she was afebrile, hemodynamically stable, pale, with a positive Trousseau sign. The electrocardiogram showed a short QT interval (Figure 1). Severe hypocalcemia was confirmed with arterial blood gas analysis (Table 1). The patient was admitted for management and further diagnostic evaluation.

**Figure 1:**
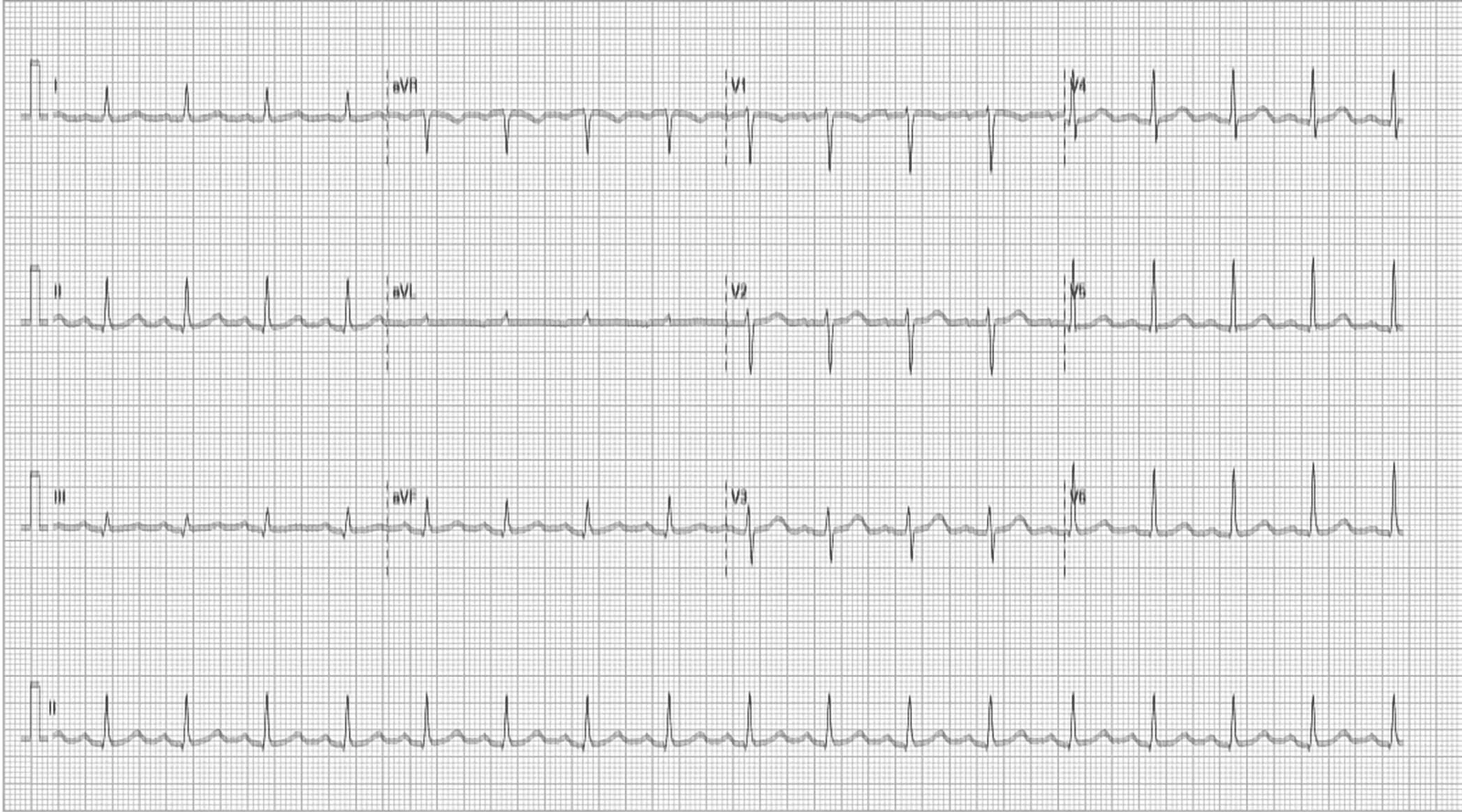
Electrocardiogram performed in the emergency department. EKG settings: 25 mm/s, 10 mm/mV, 50 Hz. The EKG shows sinus rhythm, heart rate 101 bpm, no atrioventricular block, QRS duration <0.1 s, no ST-segment or T-wave abnormalities, short corrected QT interval (QTc 420 ms), and normal axis.

**Table 1:** Arterial blood gas analysis on admission

Parameter	Result	Reference range
pH	7.43	7.35–7.45
pCO₂ (mmHg)	32.0	35–45
pO₂ (mmHg)	72.0	75–100
HCO₃⁻ (mmol/L)	25	22–26
Ionized calcium, Ca²⁺ (mmol/L)	0.64	1.12–1.30
Sodium, Na⁺ (mmol/L)	137	135–145
Potassium, K⁺ (mmol/L)	3.9	3.5–5.0
Chloride, Cl⁻ (mmol/L)	120	98–108

Laboratory findings on admission are summarised in Table 2. They demonstrated normocytic normochromic anemia and thrombocytopenia, with no evidence of hemolysis, iron deficiency, or vitamin B12 or folate deficiency. Severe hypocalcemia and hyperphosphataemia were present, while other electrolytes, creatinine and uric acid levels were within the reference range. Parathyroid hormone (PTH) levels were within the reference range, whereas 25-hydroxyvitamin D levels were insufficient. Thyroid function tests were normal. Liver enzymes were unremarkable, except for a markedly elevated alkaline phosphatase level. Lactate dehydrogenase was also elevated.

**Table 2:** Summary of laboratory results upon admission

Parameters	Values	SI Reference
Haemoglobin	10.0 g/dL	Female: 12.0–16.0 g/dL
Mean corpuscular volume (MCV)	90 fL	80–100 fL
Platelet count	96 x 10^9^/L	150–400 x 10^9^/L
White blood count (WBC)	6.5 x 10^9^/L	4.5–11.0 x 10^9^/L
Urea	6 mmol/L	2.5–6.4 mmol/L
Creatinine	62 µmol/L	53–106 µmol/L
Sodium (Na^+^)	140 mmol/L	136–146 mmol/L
Potassium (K^+^)	4.0 mmol/L	3.5–5.0 mmol/L
Magnesium (Mg^2+^)	0.75 mmol/L	0.75–1.0 mmol/L
Phosphorus (inorganic)	2.58 mmol/L	1.0–1.5 mmol/L
Adjusted calcium	1.17 mmol/L	2.1–2.6 mmol/L
Albumin	40 g/L	35–55 g/L
Alkaline phosphatase (ALP)	1667 U/L	25–100 U/L
Creatine kinase (CK)	70 U/L	Female: 10–70 U/L
Lactate dehydrogenase	513 U/L	45-200 U/L
Uric acid	0.40 mmol/L	0.18–0.48 mmol/L
Thyroxin-stimulating hormone (TSH)	0,572 mIU/L	0.4–4.0 mIU/L
Free T_4_	19,18 pmol/L	12.0–21.9 pmol/L
Intact parathyroid hormone (PTH)	30,83 ng/L	10–60 ng/L
25-hydroxyvitamin D	60 nmol/L	Deficiency: < 50 nmol/L Insufficiency: 50–72 nmol/L

Review of previous medical records revealed persistent, untreated hypocalcemia since two years after total thyroidectomy, with progressive worsening over time. Low ionized calcium levels had also been documented during the two previous ED visits but had been overlooked.

A CT scan revealed diffuse osteosclerosis throughout the vertebral column, with a heterogeneous pattern in the lumbosacral region. Overall, these findings raised suspicion for osteoblastic lesions. No pathological fractures were identified (Figure 2).

**Figure 2:**
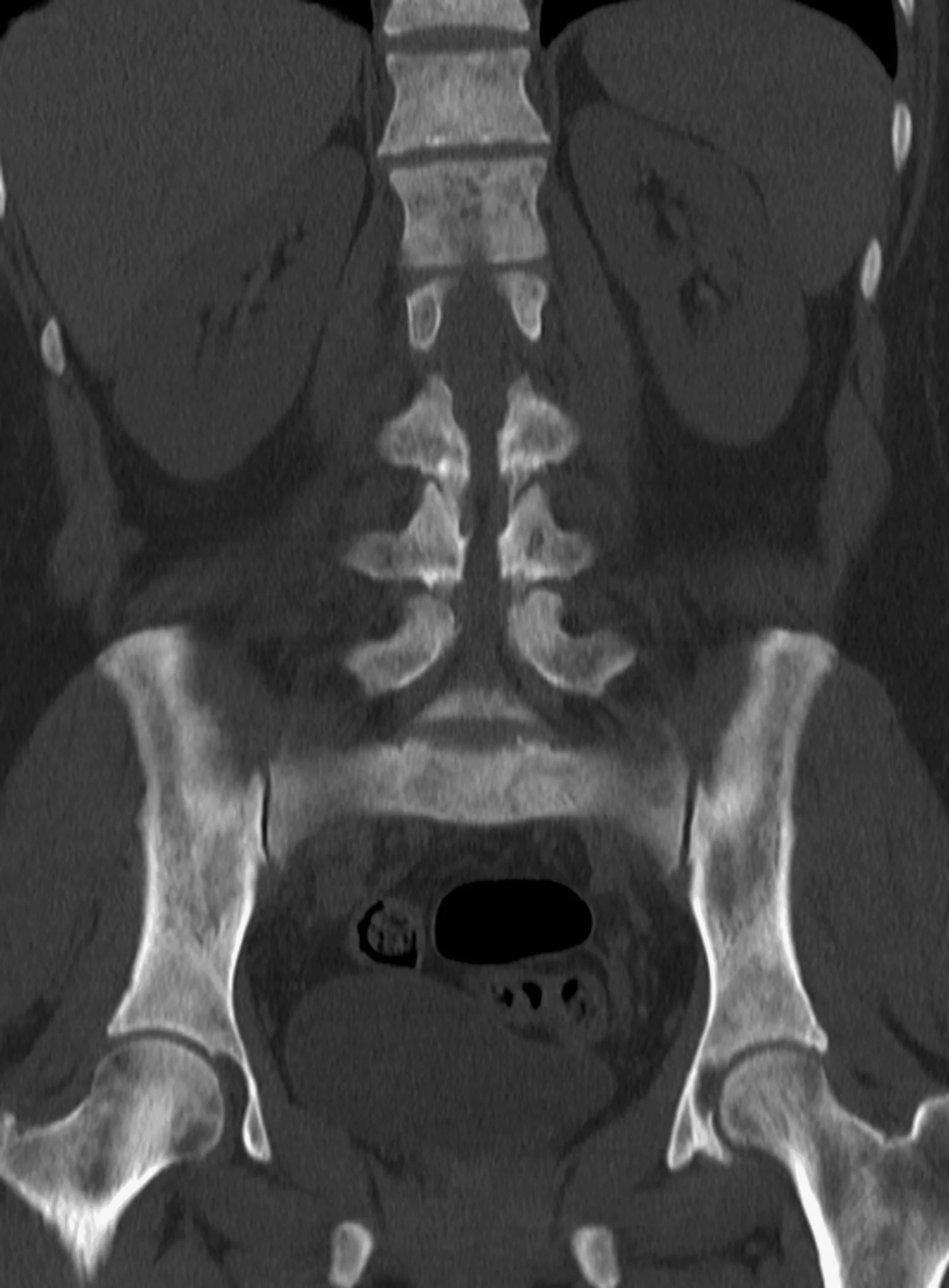
Computed tomography (CT) of the lumbosacral spine Coronal CT reconstruction of the lumbosacral spine and pelvis demonstrating diffuse, heterogeneous osteosclerosis involving the lumbar vertebral bodies, sacrum, and iliac wings.

An extensive investigation for an occult primary malignancy, including cranial, thoracic and abdominopelvic CT (Figure 3), thyroid and breast imaging (Figure 4-5, respectively), failed to identify the primary tumor. Upper gastrointestinal endoscopy showed no mucosal abnormalities (Figure 6). Biopsies were obtained from the gastric antrum and body, with no muscularis mucosae represented in the samples. All fragments exhibited a mild lymphoplasmacytic inflammatory infiltrate in the lamina propria. No atrophy or intestinal metaplasia was observed. Colonoscopy revealed no significant endoscopic abnormalities, although visualization was limited by inadequate bowel preparation (Figure 7).

**Figure 3:**
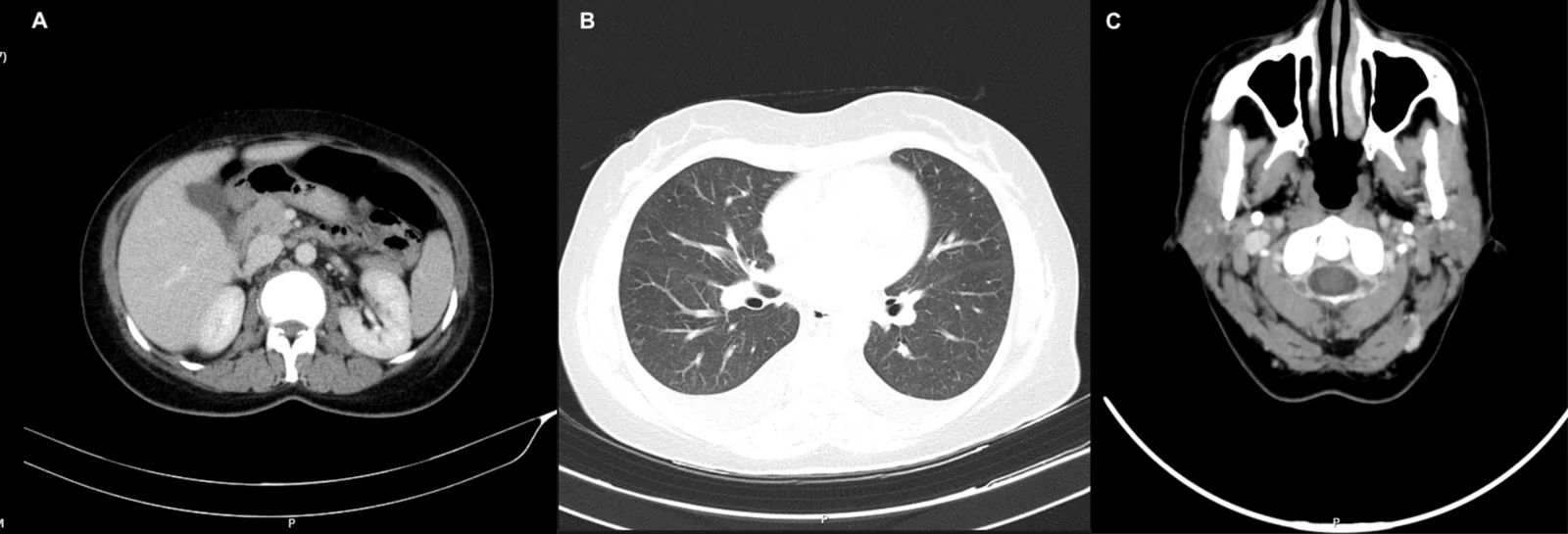
Multiregional CT imaging in the search for an occult primary malignancy. A: Abdominal CT: The liver was normal in size and contour, without steatosis or focal lesions. The spleen was normal, with no lymphadenopathy or free intraperitoneal fluid; B: Thorax CT: No suspicious pulmonary abnormalities, consolidations, or space-occupying lesions were identified. No hilar or mediastinal lymphadenopathy was observed. Minimal bilateral pleural effusions were present; C: Cranial CT: No focal abnormalities of brain parenchymal density or morphology were identified, with no evidence of an intracranial space-occupying lesion.

**Figure 4:**
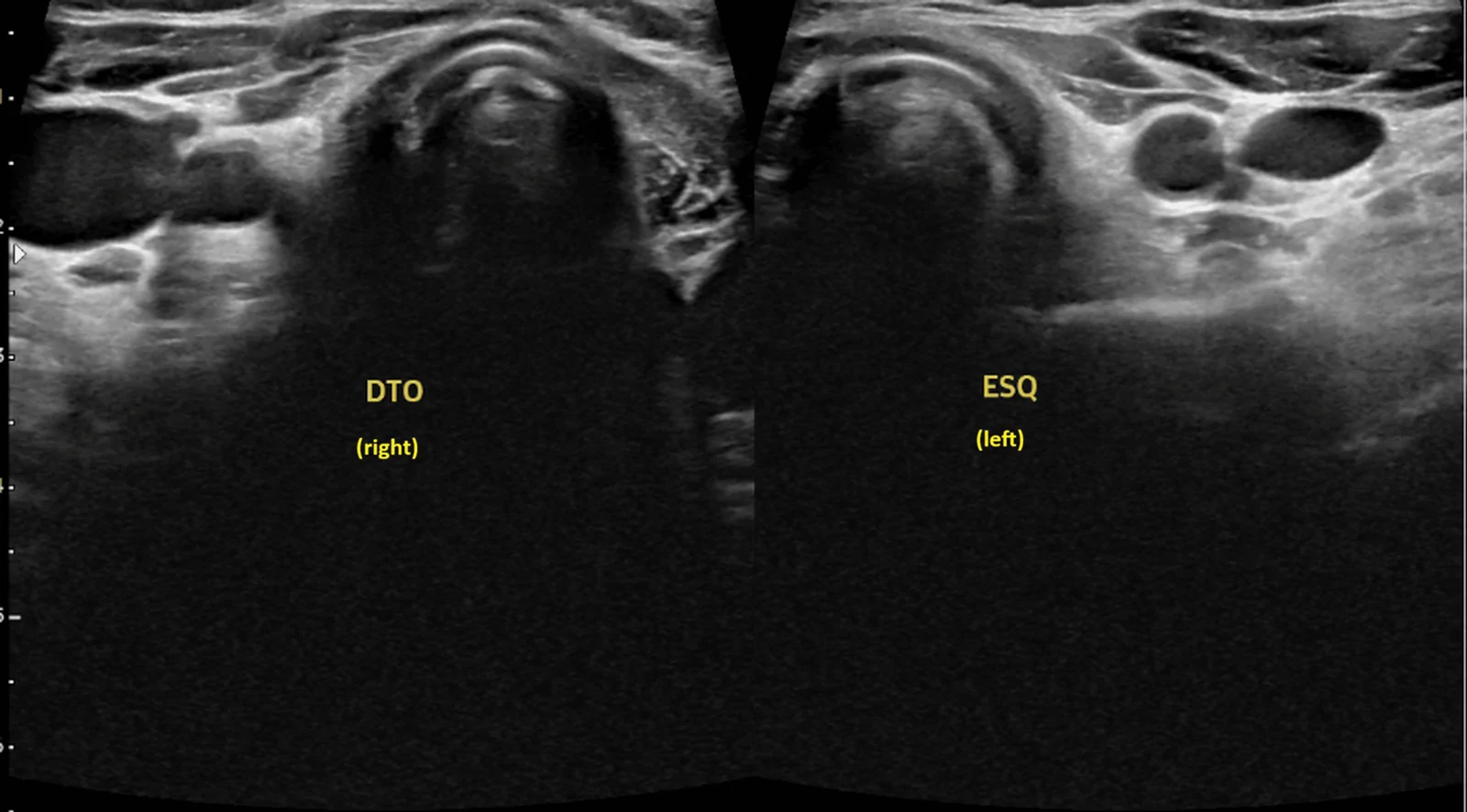
Thyroid bed ultrasound in the evaluation of an occult primary malignancy. Thyroid ultrasound demonstrating right and left thyroid beds, showing no sonographic evidence of suspicious lesions or neoplastic disease.

**Figure 5:**
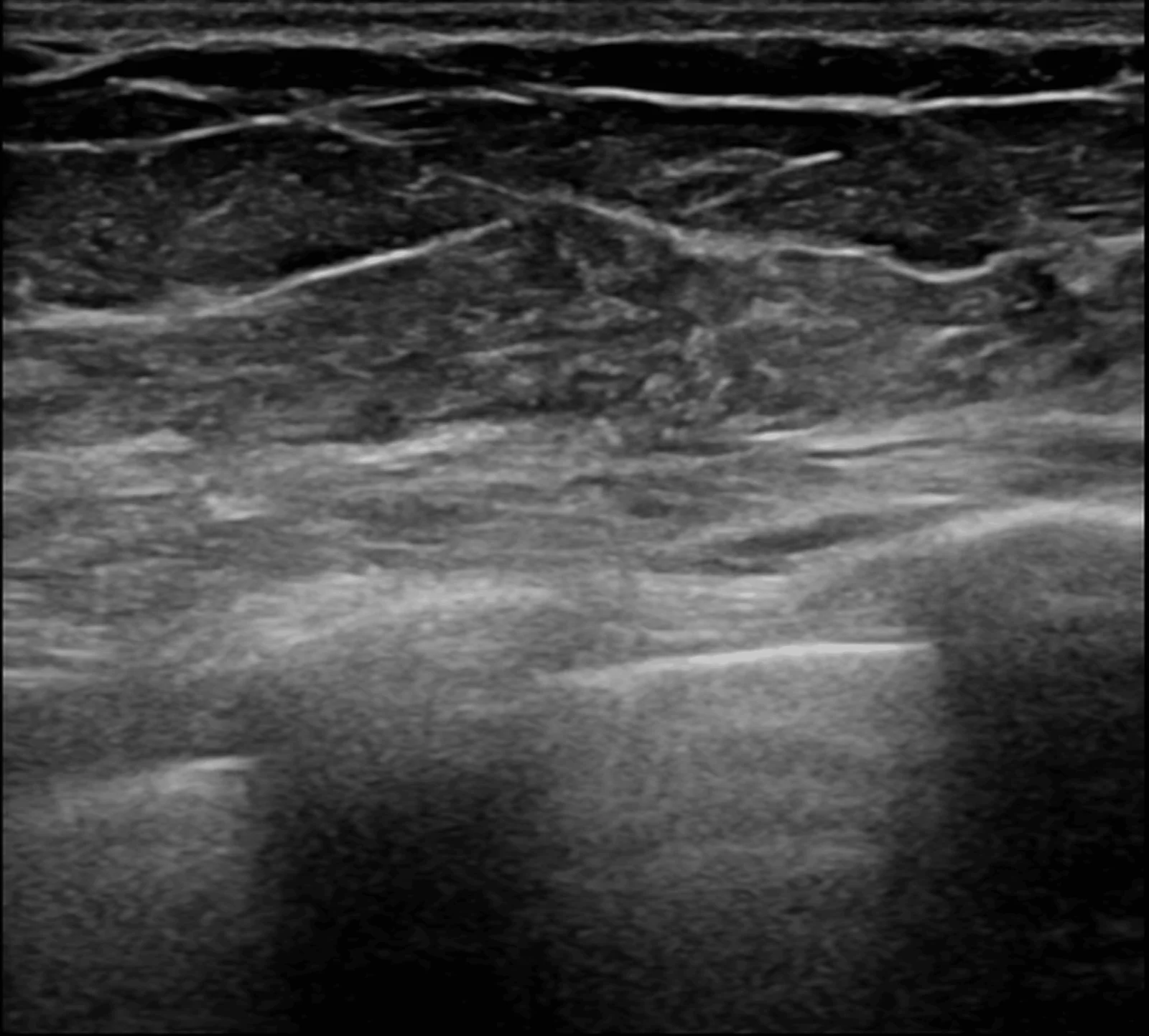
Breast ultrasound in the evaluation of an occult primary malignancy. Representative ultrasound image showing normal-appearing breast parenchyma, with no suspicious focal lesion identified.

**Figure 6:**
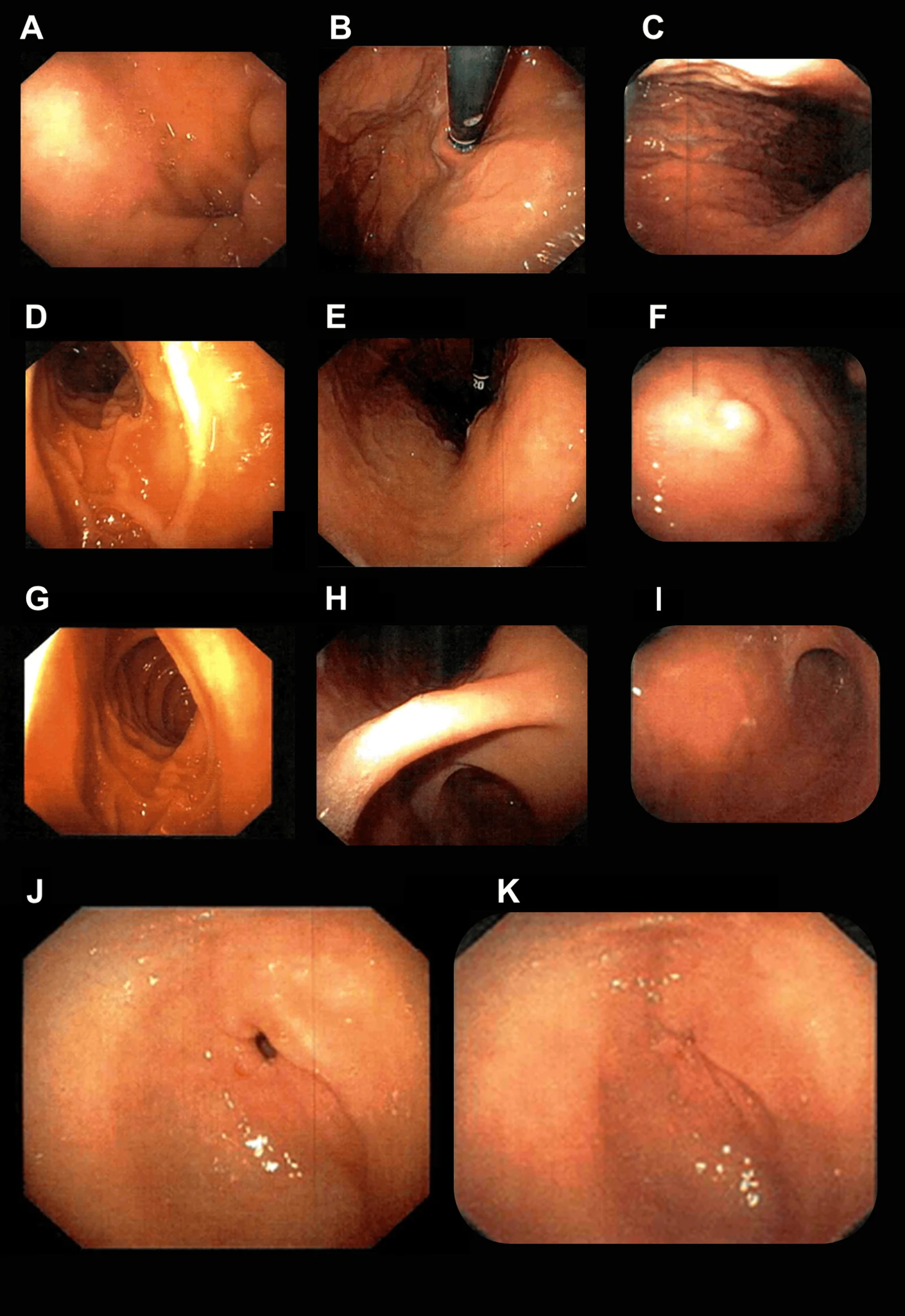
Upper gastrointestinal endoscopy in the evaluation of an occult primary malignancy. Representative endoscopic images showing grossly unremarkable mucosa of the oesopaghus, stomach and duodenum. A: Duodenal bulb; B: Cardia; C and I: Oesopaghus (proximal and distal, respectively); D and G: Second part of the duodenum; E: Lesser curvature; F: Fundus; H: Incisura angularis; J and K: Antrum

**Figure 7:**
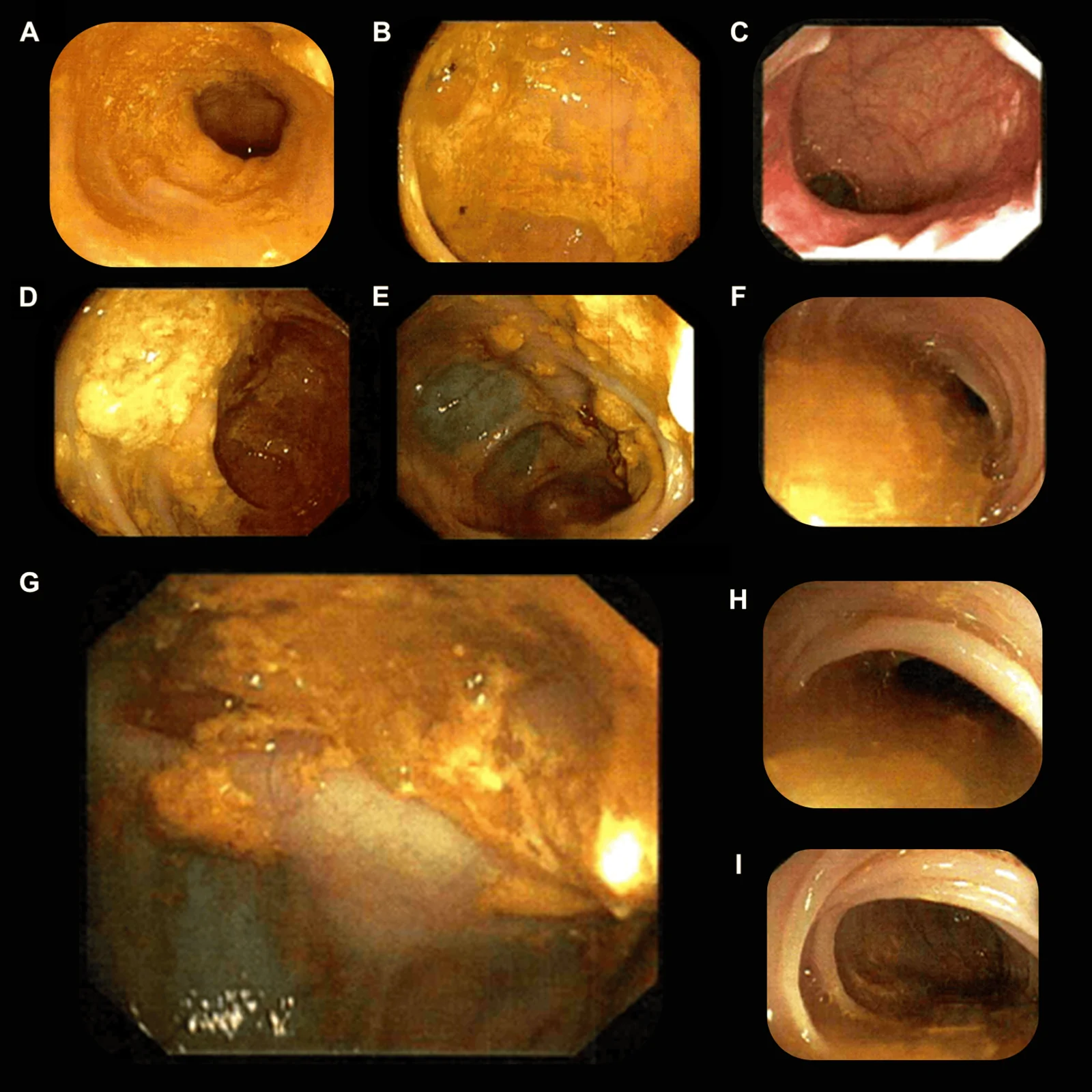
Colonoscopy in the evaluation of an occult primary malignancy. Representative colonoscopic images of the terminal ileum and colonic segments, showing grossly unremarkable mucosa in the visualized areas. A: Terminal ileum; B: Cecum; C: Rectum; D and E: Ascending colon (proximal and distal, respectively); F: Sigmoid colon; G: Hepatic flexure; H and I: Descending colon.

During hospitalization, the patient developed progressive worsening of the anemia and thrombocytopenia, along with new-onset leucopenia. Coagulopathy with hypofibrinogenaemia also developed, accompanied by spontaneous hematomas involving the upper and lower limbs without mucosal bleeding. Hematological and coagulation findings are summarised in Table 3. Peripheral blood film demonstrated a leucoerythroblastic picture, characterized by mild anisocytosis, polychromasia, rare dacrocytes, nucleated red blood cells (8 per 100 leucocytes), and a left-shifted myeloid series with 3% blasts, 1% myelocytes, and 5% metamyelocytes; no Auer rods were identified. 

**Table 3:** Hematological and coagulation findings during hospitalization.

Parameter	Result	Reference range
Haemoglobin	7.0 g/dL	Female: 12.0–16.0 g/dL
Platelet count	22 × 10⁹/L	150–400 × 10⁹/L
White blood cell count	2.7 × 10⁹/L	4.5–11.0 × 10⁹/L
Prothrombin time (PT)	16.5 s	11–15 s
Activated partial thromboplastin time (aPTT)	28s	25–40 s
Fibrinogen	1.28 g/L	2.0–4.0 g/L

Bone marrow evaluation was subsequently performed. Aspiration was challenging, yielding a nearly dry tap. The results of flow cytometry and reverse-transcription polymerase chain reaction (RT-PCR) for the *PML::RARa* fusion gene are summarised in Table 4.

**Table 4:** Bone marrow aspirate findings

Investigation	Results	Interpretation
Flow cytometry. Markers: CD34, CD117, CD36, CD71, CD3, CD4, CD5, CD8, CD45, CD56, CD19, CD20, CD38, κ / λ	0.86% blasts. No aberrant dysplastic phenotypes. Normal T, B and NK cell phenotypes.	Acute leukaemia not supported
RT-PCR t(15;17)(q22;q21).	*PML::RARa* not detected	Acute promyelocytic leukaemia not supported

The whole-body positron emission tomography/computed tomography scan with Fluorine-18 Fluorodeoxyglucose (18F-FDG PET/CT) showed diffuse and heterogeneous increased metabolic activity in the bone and bone marrow throughout the axial and appendicular skeleton, raising suspicion for malignant involvement. Increased splenic metabolic activity was also observed, although this finding was nonspecific (Figure 8). However, subsequent abdominal MRI did not confirm splenic involvement.

**Figure 8:**
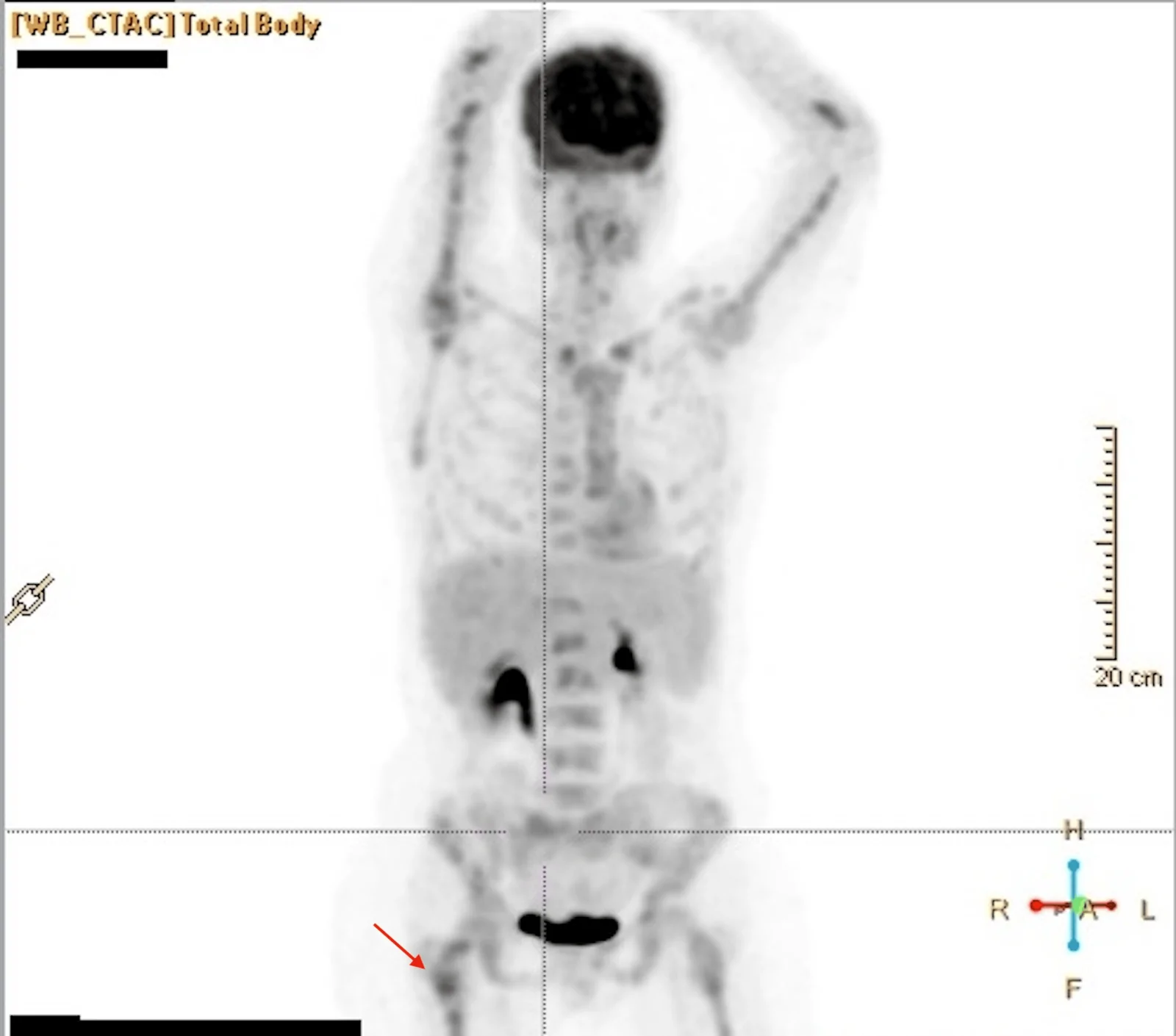
Whole-body 18F-FDG PET/CT in the search for an occult primary malignancy. Diffuse ¹⁸F-FDG uptake throughout the axial and appendicular skeleton, demonstrating a heterogeneous distribution and diffuse sclerosis; the maximum standardized uptake value (SUVmax) was 6.2 in the right femoral intertrochanteric region (arrow).

Finally, bone marrow biopsy revealed an extensive infiltration by a poorly differentiated adenocarcinoma with numerous signet-ring cells, positive for CK CAM5.2 and CDX2, with partial and heterogeneous expression of CK7, and negative for CK20 with pronounced stromal fibrosis, suggesting signet-ring cell gastric adenocarcinoma (Figure 9-12).

**Figure 9:**
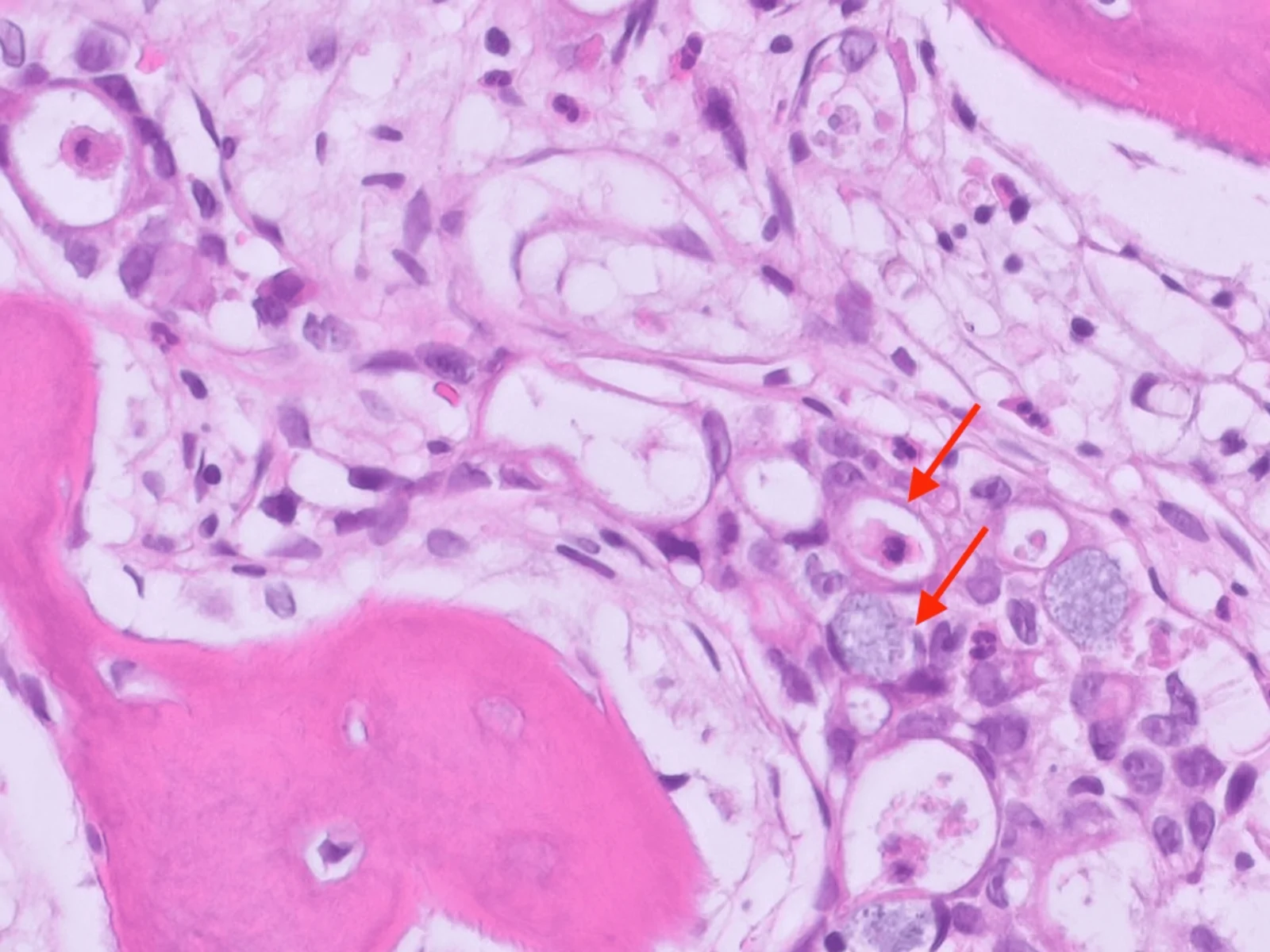
Histopathological evaluation of the bone marrow biopsy. Hematoxylin–Eosin staining demonstrates trabecular bone with stromal fibrosis and infiltration by atypical glandular structures (arrows). Magnification: 40×.

**Figure 10:**
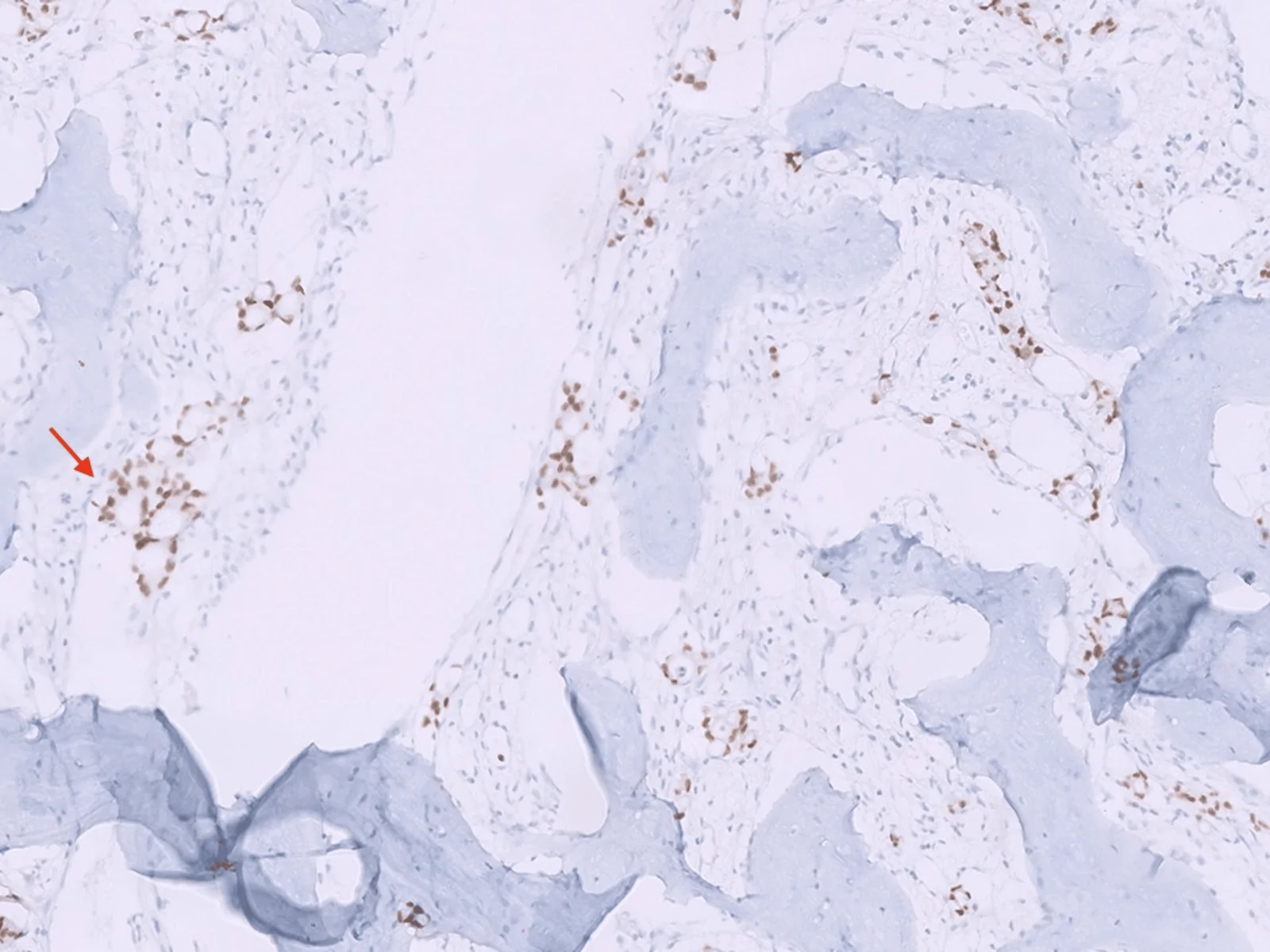
Histopathological and immunohistochemical evaluation of the bone marrow biopsy (CDX2 staining). Immunohistochemical staining for CDX2 demonstrates expression in the neoplastic glandular cells (arrow). Magnification: 10×.

**Figure 11:**
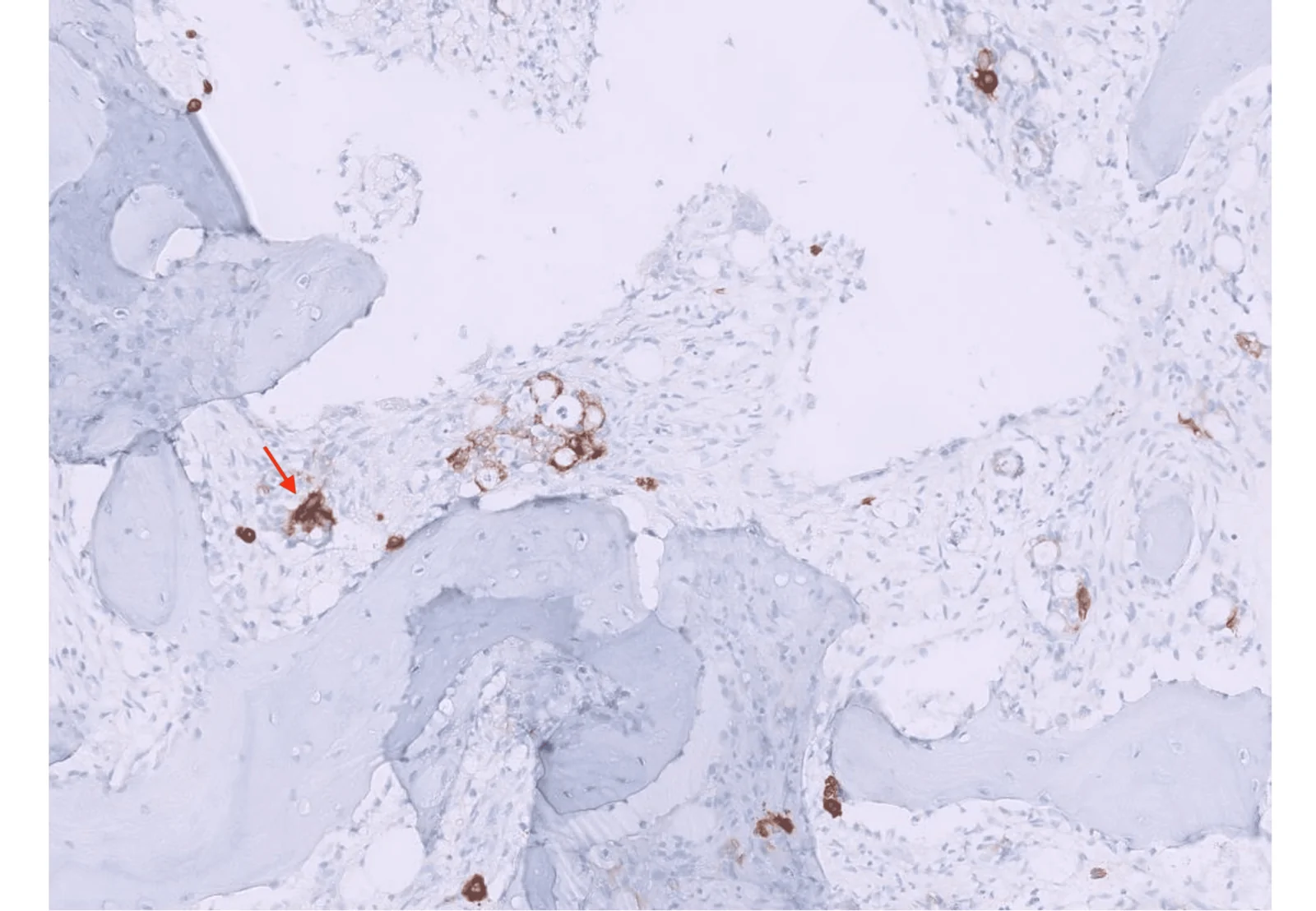
Histopathological and immunohistochemical evaluation of the bone marrow biopsy (CK7 staining). Immunohistochemical staining for cytokeratin 7 (CK7) demonstrates partial and heterogeneous expression in the atypical epithelial cells (arrow). Magnification: 10×.

**Figure 12:**
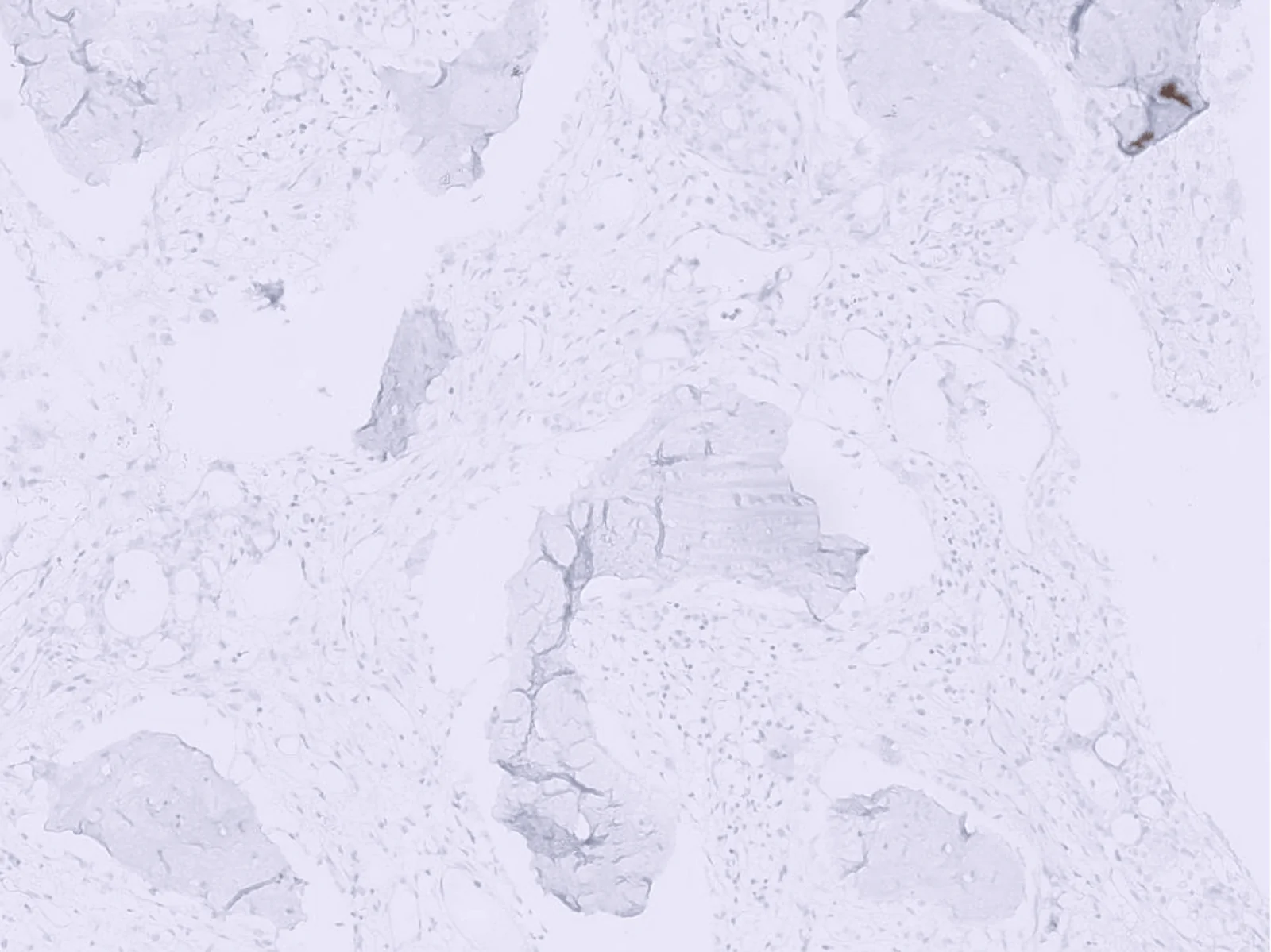
Histopathological and immunohistochemical evaluation of the bone marrow biopsy (CK20 staining). Immunohistochemical staining for cytokeratin 20 (CK20) shows no immunoreactivity in the infiltrating cells. Magnification: 10×.

During hospitalization, the patient required multiple transfusions of packed red blood cells and platelet concentrates. Despite intensive replacement with calcium, calcitriol and cholecalciferol, hypocalcemia remained difficult to control. Her clinical status progressively deteriorated, and she died shortly after the diagnosis was established.

## Discussion

We present a patient with severe symptomatic hypocalcemia and anemia, which rapidly progressed to pancytopenia, ultimately leading to the diagnosis of signet-ring cell gastric adenocarcinoma. The diagnosis was challenging because of the patient's atypical clinical presentation. Tumor Lysis Syndrome (TLS) was considered due to hypocalcemia, hyperphosphataemia and elevated LDH, but the absence of hyperuricemia, hyperkalemia and acute kidney injury made this diagnosis unlikely [4]. Pancytopenia and coagulopathy with fibrinogen consumption raised suspicion for Acute Myeloid Leukemia (AML), particularly Acute Promyelocytic Leukemia (APL). The combination of rapidly progressive pancytopenia and leukoerythroblastosis also prompted consideration of primary myelofibrosis and bone marrow infiltration by a solid tumor. Bone marrow biopsy ultimately established the diagnosis of metastatic signet-ring cell gastric adenocarcinoma.

Signet-ring cell carcinoma spreads predominantly within the lamina propria and submucosa, making tumor margins difficult to assess and superficial biopsies prone to false-negative results [1,5]. In our patient, the absence of muscularis mucosa sampling may explain the inconclusive endoscopy [5]. Furthermore, PET/CT failed to detect the gastric tumour because of the low FDG avidity characteristic of the signet-ring cell gastric carcinoma [6]. 

Symptomatic hypocalcemia in malignancy is extremely uncommon, affecting 1.6% of oncological patients [7]. It is most frequently associated with osteoblastic metastases [7], a phenomenon well described in prostate [8], but less frequently reported in gastric cancer [8,9]. The underlying mechanism involves increased calcium uptake from the bloodstream due to heightened osteoblastic activity [8-10]. However, osteoblastic metastases alone rarely cause significant hypocalcemia, as compensatory mechanisms usually maintain calcium homeostasis [10]. In this case, two additional factors likely contributed to the development of malignancy-associated hypocalcemia [11]. The patient had previously undergone total thyroidectomy, which may have resulted in inadvertent parathyroid injury. In fact, hypocalcemia had been documented two years after thyroidectomy and progressively worsened thereafter. Furthermore, the patient had normal PTH levels despite the presence of hypocalcemia, indicating an inadequate parathyroid response [12,13]. Another contributing factor was the patient's insufficient 25-hydroxyvitamin D levels, a condition highly prevalent in cancer patients [11].

Bone metastases from gastric cancer are rare, with an incidence of 4.18% [14]. Although the pathophysiological mechanisms remain incompletely understood [15], angiogenesis, a key factor in gastric carcinogenesis and tumor progression, is believed to contribute to the development of bone metastasis [16]. Bone involvement occurs more frequently in younger patients [14] and in poorly differentiated subtypes [14], as in the present case. Markedly elevated LDH and alkaline phosphatase levels in this case suggested increased bone turnover, supporting bone and bone marrow involvement [3].

Bone marrow metastases from solid tumors are uncommon and more commonly reported in prostate, breast and lung cancer. Conversely, they are exceedingly rare in gastric cancer (0.9%) [3]. When present, they are usually linked to poorly differentiated tumors [3,17] and signet-ring cell carcinoma [3,17]. It is more frequently reported in younger patients, with a median age of 47 years [17]. Bone marrow infiltration commonly presents with thrombocytopenia but may also cause pancytopenia owing to marrow replacement by tumor cells, as illustrated by the leukoerythroblastic reaction in our patient [3,18].

The prognosis of gastric cancer with either bone [15] or bone marrow metastases [3,17] is extremely poor. Patients with bone marrow involvement have a reported median survival of only 44 days [17]. Consistent with these observations, our patient's clinical condition deteriorated rapidly, and she died shortly after the diagnosis was established.

To the best of our knowledge, this is the first clinical report of gastric adenocarcinoma presenting simultaneously with hypocalcemia secondary to osteoblastic metastases and pancytopenia resulting from bone marrow infiltration.

Learning points

Signet-ring cell gastric adenocarcinoma may evade conventional diagnostic investigations, as superficial endoscopic biopsies may yield false-negative results; severe hypocalcemia may rarely be the initial manifestation of gastric adenocarcinoma with osteoblastic bone metastases; Bone and bone marrow metastases are rare manifestations of gastric adenocarcinoma, typically occurring in poorly differentiated tumors and carrying a poor prognosis; Bone marrow metastases may present with pancytopenia with leucoerythroblastic reaction, resembling haematologic malignancies.

## Conclusions

This case highlights the diagnostic complexity of signet-ring cell gastric adenocarcinoma. Recognizing the rare constellation of severe hypocalcemia, osteoblastic bone metastases, and leucoerythroblastic pancytopenia may serve as an important diagnostic clue to an underlying solid malignancy, even when the primary site is not initially apparent. Although uncommon, this presentation should prompt consideration of metastatic gastric adenocarcinoma in the differential diagnosis, particularly when initial investigations are inconclusive. Early recognition of these atypical features may facilitate timely diagnostic evaluation and appropriate multidisciplinary management.
